# Discrepancy between cystatin C-based and creatinine-based eGFR predicts all-cause mortality in a community-based population: the Takahata study

**DOI:** 10.1007/s10157-026-02853-6

**Published:** 2026-03-27

**Authors:** Takaya Suzuki, Kazunobu Ichikawa, Natsuko Suzuki, Masafumi Watanabe, Tsuneo Konta

**Affiliations:** 1 Department of Cardiology, Pulmonology, and Nephrology, Yamagata University School of Medicine, Yamagata, Japan; 2 Department of Public Health and Hygiene, Yamagata University Graduate School of Medical Science, 2-2-2, Iida-Nishi, Yamagata, 990-9585 Japan; 3 Institute for Promotion of Medical Science Research, Yamagata University Faculty of Medicine, Yamagata, Japan

**Keywords:** Cystatin C, Creatinine, Estimated GFR, Chronic kidney disease, General population

## Abstract

**Background:**

Discrepancies between the cystatin C-based estimated glomerular filtration rate (eGFRcys) and the creatinine-based estimated glomerular filtration rate (eGFRcr) have been linked to adverse outcomes in Western populations. However, their prognostic significance in community-dwelling Japanese individuals remains unclear. Therefore, we conducted a prospective cohort study to evaluate this association.

**Methods:**

We analyzed data from 1308 participants who underwent simultaneous measurements of serum creatinine and cystatin C levels between 2004 and 2006, with a median follow-up of 18.5 years. The discrepancy (eGFRdiff = eGFRcys − eGFRcr) was categorized into three groups: a low group (< − 10 mL/min/1.73 m^2^), a reference group (− 10 to 10 mL/min/1.73 m^2^), and a high group (≥ 10 mL/min/1.73 m^2^). All-cause and cardiovascular mortality were assessed using Kaplan–Meier curves, Cox proportional hazards models, and restricted cubic spline analyses.

**Results:**

During the follow-up period, 386 participants (29.5%) died, including 120 cardiovascular-related deaths. Compared with the reference group, individuals in the eGFRdiff < − 10 group exhibited a significantly higher risk of all-cause mortality (hazard ratio [HR]: 1.41; 95% confidence interval [CI]: 1.10–1.80), whereas those in the ≥ 10 group showed a lower risk (HR: 0.56; 95% CI: 0.33–0.97). No significant association was observed with cardiovascular mortality. Incorporating eGFRdiff into the baseline models improved discrimination for both all-cause and cardiovascular mortality.

**Conclusion:**

The discrepancy between eGFRcys and eGFRcr levels is an independent predictor of all-cause mortality in a community-based Japanese population. eGFRdiff may serve as a simple yet informative marker for identifying individuals at increased risk of death.

**Supplementary Information:**

The online version contains supplementary material available at 10.1007/s10157-026-02853-6.

## Introduction

Chronic kidney disease (CKD) is a significant public health concern owing to its close association with cardiovascular disease and all-cause mortality [1]. Therefore, early detection and accurate risk assessment are essential for the effective management of CKD. Estimated glomerular filtration rate (eGFR) is widely used to assess kidney function, and in Japan, population-specific equations developed by the Japanese Society of Nephrology are widely used for these estimations [2, 3].

However, serum creatinine levels are influenced by several non-GFR-related factors, including muscle mass, nutritional status, age, and ethnicity, which may result in overestimation or underestimation of kidney function, particularly in specific populations [4]. In contrast, cystatin C is less affected by muscle mass and is considered to more accurately reflect kidney function in such cases [5]. The 2024 Kidney Disease: Improving Global Outcomes guidelines recommend using either cystatin C-based estimated glomerular filtration rate (eGFRcys) or a combined creatinine–cystatin C formula (eGFRcr-cys) in clinical situations where eGFRcr may be unreliable [6]. Importantly, cystatin C, either alone or in combination with creatinine, has been demonstrated to improve the accuracy of GFR estimation and to enhance the prognostic ability for outcomes such as mortality and progression to end-stage renal disease (ESRD) [7, 8].

Recent studies have highlighted the clinical implications of discrepancies between eGFRcys and eGFRcr, referred to as eGFRdiff (eGFRcys − eGFRcr). A cross-sectional study of older residents in a specific region of the United States reported that nearly two-thirds of participants exhibited an eGFR difference exceeding 10%, and approximately 20% exhibited an eGFR difference greater than 30% [9]. Furthermore, according to the Chronic Renal Insufficiency Cohort (CRIC) study conducted on patients with chronic kidney disease (CKD) in the United States, an eGFRdiff below − 15 mL/min/1.73 m^2^ is associated with a significantly increased risk of all-cause mortality and end-stage renal disease [10]. Additionally, it has been proposed that eGFRdiff may reflect poor systemic health conditions such as frailty and sarcopenia. In this context, eGFRcys value below eGFRcr value is associated with worse outcomes, whereas an eGFRcys value exceeding the eGFRcr value may indicate better overall health and favorable prognosis [11].

Although these findings from Western populations are informative, the prognostic implications of eGFRdiff in community-dwelling Japanese individuals remain largely unknown. Given that the equations used for eGFR estimation and demographic factors such as body composition, diet, and lifestyle differ between Japanese and Western populations [6], independent validation is necessary. Previous cohort studies of Japanese adults have reported that eGFRcys is a more accurate predictor of mortality than eGFRcr [12]. Moreover, substantial discrepancies between the two measures have been associated with frailty and reduced skeletal muscle mass [13].

However, no prospective, long-term study has specifically evaluated the relationship between eGFRdiff and all-cause mortality in the general Japanese population. Therefore, this study aimed to investigate the long-term association between eGFRdiff and all-cause mortality in a well-characterized cohort of community-dwelling Japanese individuals. We aimed to explore the clinical significance of eGFR discrepancies within the Japanese context.

## Materials and methods

### Study population

This study was conducted as part of an ongoing molecular epidemiology project that utilized resources from the 21 st Century Center of Excellence (COE) Program and Global COE Program in Japan. It was a prospective cohort study design, with baseline data collected between 2004 and 2006, and participants were followed up at 5-year intervals. Details regarding the study design, recruitment methods, and cohort characteristics have been previously described [14].

**Fig. 1:**
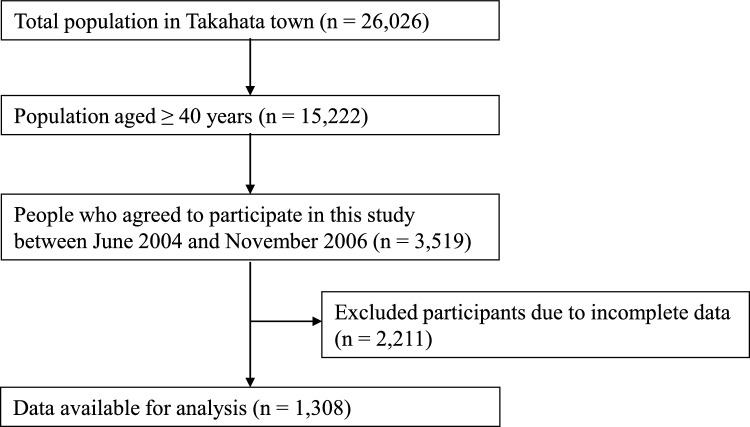
Flowchart of study participants. Flowchart illustrating the inclusion and exclusion process of the 1308 community-dwelling participants who underwent both serum creatinine and cystatin C measurements between 2004 and 2006

Among the original participants, 1,308 individuals (588 men and 720 women) were included in this analysis between 2004 and 2006. They underwent health examinations, including measurements of serum creatinine and cystatin C levels. The participants were followed up for a median of 18.5 years, with a maximum follow-up duration of 20 years. All study protocols were approved by the Institutional Ethics Committee, and written informed consent was obtained from all participants (IRB approval number: Yamagata University, 2006-1). The inclusion and exclusion criteria are summarized in Fig. 1.

### Measurement methods

At baseline, the participants completed a self-administered questionnaire assessing their medical history, medication use, and clinical symptoms. Blood pressure was measured in a seated position after at least 5 minutes of rest using a mercury sphygmomanometer. Hypertension was defined as systolic blood pressure ≥ 140 mmHg, diastolic blood pressure ≥ 90 mmHg, or current use of antihypertensive medication. BMI was calculated as weight in kilograms divided by height in meters squared (kg/m^2^), with BMI < 18.5 kg/m^2^ classified as underweight and ≥ 25.0 kg/m^2^ as overweight. Diabetes was defined as a fasting blood glucose level of ≥ 126 mg/dL, a glycated hemoglobin (HbA1c) level ≥ 6.5%, or use of antidiabetic medication. Dyslipidemia was defined as meeting at least one of the following criteria: low-density lipoprotein cholesterol (LDL-C) ≥ 140 mg/dL, high-density lipoprotein cholesterol (HDL-C) < 40 mg/dL, triglycerides ≥ 150 mg/dL, non-HDL-C ≥ 170 mg/dL, or use of lipid-lowering drugs. Urine albumin-to-creatinine ratio (UACR) was measured using a single-spot morning urine sample.

Urinary albumin levels were measured using an immunoturbidimetric assay. Albuminuria was defined as UACR ≥ 30 mg/gCr, and overt albuminuria as ≥ 300 mg/gCr [15]. Dipstick urinalysis was performed, with results interpreted by physicians or trained assistants. Overt proteinuria was defined as a dipstick result of ≥ 1+. Serum creatinine was measured using an enzymatic method and serum cystatin C levels were measured using a latex-enhanced immunonephelometric assay. eGFRcr and eGFRcys were calculated using the Japanese Society of Nephrology equations, which have been validated in the Japanese population [2, 3].

### Outcome assessment

Vital status and causes of death were obtained annually from official death certificates maintained by the municipal government. All-cause mortality was defined as death from any cause. Cardiovascular mortality was defined as deaths corresponding to ICD-10 codes I00-I99. Participants were followed from baseline (2004–2006) until death or December 31, 2023.

### Definition of eGFR discrepancy

eGFR discrepancy (eGFRdiff) was calculated as the difference between eGFRcys and eGFRcr, using the formula: eGFRdiff = eGFRcys − eGFRcr [10]. For the primary analysis, participants were classified into three groups based on their eGFRdiff values: a low group (< − 10 mL/min/1.73 m^2^), a reference group (− 10 to 10 mL/min/1.73 m^2^), and a high group (≥ 10 mL/min/1.73 m^2^). These cut-off values were rounded to the nearest integer to enhance clinical interpretability. The distribution of eGFRdiff showed a mean of − 4.9 ± 14.8 mL/min/1.73 m^2^ and a median of − 4.55 (interquartile range: − 13.8 to +4.55), exhibiting a left-skewed distribution. For the primary cutoff values, we adopted ±10 mL/min/1.73 m^2^. This value was chosen because it corresponds to approximately one standard deviation from the mean, is close to the interquartile range, and is an integer that is easy to interpret clinically.

### Statistical analysis

Continuous variables were expressed as mean ± standard deviation (SD) or as median with interquartile range (IQR), and categorical variables were presented as counts and percentages. Group comparisons were conducted using one-way analysis of variance (ANOVA) or unpaired *t*-tests for continuous variables, and chi-square tests for categorical variables.

Survival curves for all-cause and cardiovascular mortality were generated using the Kaplan-Meier method and compared using the log-rank test. Cox proportional hazards models were employed to estimate the hazard ratios (HRs) and 95% confidence intervals (CIs) for mortality outcomes. Multivariate models were adjusted for potential confounders, including age, sex, BMI, eGFRcr, alcohol consumption, smoking status, hypertension, diabetes, dyslipidemia, and albuminuria.

Furthermore, to assess the robustness of the findings, sensitivity analyses were conducted using quartile-based classifications of eGFRdiff and a restricted cubic spline model with eGFRdiff = 0 mL/min/1.73 m^2^ as the reference value. To visualize adjusted survival probabilities, model-based adjusted survival curves were generated using Cox regression with covariates fixed at representative values obtained by the standardization method and compared using the likelihood ratio test. In these analyses, the covariates were consistent with those used in the multivariate Cox proportional hazards. To assess the discriminatory ability of the predictive models, we calculated the Integrated Discrimination Improvement (IDI) with and without the inclusion of eGFRdiff in the baseline model.

Statistical significance was set at a two-tailed *p*-value < 0.05. All statistical analyses were performed using JMP Pro, version 18 (SAS Institute Inc., Cary, NC, USA) and R, version 4.3.2 (R Foundation for Statistical Computing, Vienna, Austria). Graphs and figures were created using GraphPad Prism, version 10.5.0 (GraphPad Software, La Jolla, CA, USA) and Adobe Illustrator, version 28.4.1 (Adobe Systems Inc., San Jose, CA, USA).

## Results

### Baseline characteristics

The baseline characteristics of the 1,308 participants with serum creatinine and cystatin C measurements are summarized in Table 1. Among them, 588 (45.0%) were men. A total of 405 participants (31.0%) reported active smoking status, and 533 (40.8%) reported habitual alcohol consumption. The prevalence rates of hypertension, diabetes, and dyslipidemia were 43.0% (n = 563), 5.6% (n = 69), and 45.4% (n = 594), respectively.

**Table 1:** Baseline Characteristics of Participants by eGFRdiff (eGFRcys − eGFRcr)

	Total	Low (< − 10)	Reference (− 10 to 10)	High (≥ 10)	*P*-value
Sample size (%)	1308	442 (33.8)	690 (52.8)	176 (13.5)	< 0.01
Age (years)	63.8 ± 9.8	67.5 ± 8.5	63.6 ± 9.6	55.7 ± 9.0	< 0.01
Male sex (%)	588 (45.0)	210 (47.5)	316 (45.8)	62 (35.2)	0.02
BMI (kg/m^2^)	23.5 ± 3.2	23.4 ± 3.3	23.5 ± 3.2	23.3 ± 3.2	0.61
Systolic blood pressure (mmHg)	134.2 ± 15.7	135.8 ± 14.7	134.2 ± 16.2	130.4 ± 15.6	< 0.01
Diastolic blood pressure (mmHg)	78.7 ± 9.9	78.6 ± 9.3	79.0 ± 9.9	78.2 ± 11.2	0.61
Smoking (%)	405 (31.0)	155 (35.1)	206 (29.9)	44 (25.0)	0.03
Alcohol consumption (%)	533 (40.8)	175 (39.6)	280 (40.6)	78 (44.3)	0.55
Hypertension (%)	563 (43.0)	207 (46.8)	295 (42.8)	61 (34.7)	0.02
Diabetes (%)	69 (5.6)	24 (5.8)	41 (6.2)	4 (2.4)	0.15
Dyslipidemia (%)	594 (45.4)	198 (44.8)	320 (46.4)	76 (43.2)	0.71
HbA1c (%)	5.2 ± 0.6	5.3 ± 0.6	5.3 ± 0.6	5.1 ± 0.6	< 0.01
Hemoglobin (g/dL)	13.7 ± 1.4	13.6 ± 1.4	13.7 ± 1.4	13.6 ± 1.6	0.25
Hematocrit (%)	41.2 ± 4.0	41.0 ± 3.9	41.4 ± 4.0	41.1 ± 4.6	0.26
Serum albumin (g/dL)	4.5 ± 0.2	4.5 ± 0.2	4.5 ± 0.2	4.6 ± 0.2	< 0.01
Uric acid (mg/dL)	5.0 ± 1.3	4.9 ± 1.3	5.1 ± 1.3	5.1 ± 1.5	0.05
hsCRP (mg/dL)	0.11 ± 0.26	0.14 ± 0.33	0.10 ± 0.23	0.06 ± 0.17	< 0.01
eGFRcr (mL/min/1.73 m^2^)	81.5 ± 17.0	90.5 ± 18.3	77.8 ± 14.6	73.3 ± 12.3	< 0.01
eGFRcys (mL/min/1.73 m^2^)	76.5 ± 16.4	69.9 ± 15.0	77.1 ± 15.0	90.9 ± 15.0	< 0.01
eGFRdiff (eGFRcys − eGFRcr)	− 4.9 ± 14.8	− 20.6 ± 9.9	− 0.6 ± 5.6	17.6 ± 7.7	< 0.01
Proteinuria (%)	51 (3.9)	18 (4.1)	31 (4.5)	2 (1.1)	0.12
Albuminuria (%)	180 (13.8)	72 (16.3)	99 (14.4)	9 (5.1)	< 0.01
UACR (mg/gCr)	9.4 (5.9–17.5)	10.9 (6.6–20.0)	9.2 (5.9–17.6)	7.2 (5.0–11.8)	0.02

Mean ± SD, n (%), median (25th–75th)

^1^Unpaired t-test, Pearson's chi-square test

*BMI* Body mass index, *eGFR* Estimated glomerular filtration rate, *hsCRP* High-sensitivity C-reactive protein, *UACR* Urine albumin indexed to creatinine

**Fig. 2:**
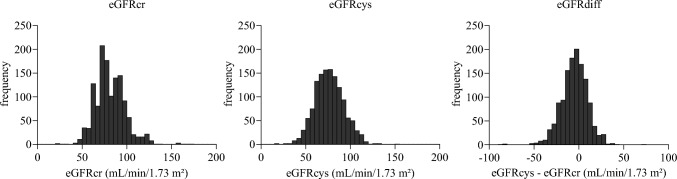
Distribution of eGFRcr, eGFRcys, and eGFRdiff. Histograms illustrating the distribution of estimated glomerular filtration rate (GFR) calculated from serum creatinine (eGFRcr), cystatin C (eGFRcys), and their difference (eGFRdiff = eGFRcys – eGFRcr), with all three variables exhibiting approximately normal distributions

The mean eGFRcr and eGFRcys were 81.5 ± 17.0 and 76.5 ± 16.4 mL/min/1.73 m^2^, respectively. Overt proteinuria (≥ 1+ on dipstick test) was observed in 51 participants (3.9%), and albuminuria (UACR ≥ 30 mg/gCr), was present in 180 participants (13.8%). The mean eGFRdiff was − 4.93 ± 14.8 mL/min/1.73 m^2^, with a median of − 4.55 (IQR: − 13.8 to 4.55), showing a mildly left-skewed distribution, similar to the distributions of eGFRcys and eGFRcr (Fig. 2).

Based on eGFRdiff, participants were categorized into three groups: the low group (< − 10 mL/min/1.73 m^2^, n = 442; 33.8%), the reference group (−10 to 10 mL/min/1.73 m^2^, n = 690; 52.8%), and the high group (≥ 10 mL/min/1.73 m^2^, n = 176; 13.5%). Participants in the low eGFRdiff group were older and exhibited higher systolic blood pressure, HbA1c levels and hsCRP (high-sensitivity C-reactive protein) than those in reference and high groups. Additionally, this group included a greater proportion of men, active smokers, individuals with hypertension, and those with overt albuminuria (Table 1).

### Kaplan–Meier analysis

**Fig. 3:**
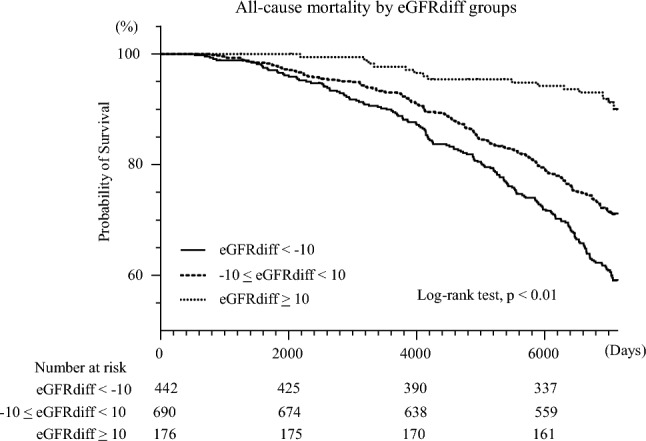
Kaplan–Meier survival curves for all-cause mortality by eGFRdiff groups. Kaplan–Meier survival curves for all-cause mortality across three eGFRdiff groups: < − 10, − 10 to 10 (reference), and ≥ 10 mL/min/1.73 m^2^, with the lowest survival observed in the group with eGFRdiff < − 10 (log-rank *p* < 0.01); eGFRdiff is defined as the difference between eGFR based on cystatin C (eGFRcys) and eGFR based on serum creatinine (eGFRcr): eGFRdiff = eGFRcys − eGFRcr

**Fig. 4:**
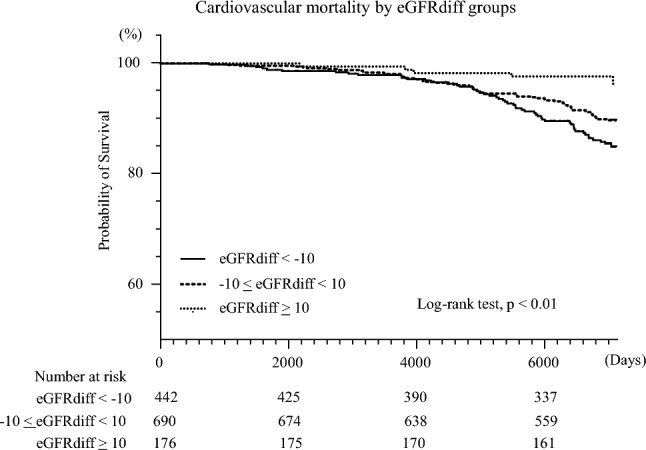
Kaplan–Meier survival curves for cardiovascular mortality by eGFRdiff groups. Kaplan–Meier survival curves for cardiovascular mortality across the eGFRdiff groups, indicating the lowest survival in the group with eGFRdiff < − 10, although intergroup differences were less pronounced than for all-cause mortality (log-rank *p* < 0.01); eGFRdiff is defined as the difference between eGFR based on cystatin C (eGFRcys) and eGFR based on serum creatinine (eGFRcr): eGFRdiff = eGFRcys − eGFRcr

Over a median follow-up period of 18.5 years, 386 participants (29.5%) died, of whom 120 (31.1% of all deaths) deaths were attributed to cardiovascular causes. Kaplan–Meier survival analysis revealed significant differences in all-cause mortality among the three eGFRdiff groups (log-rank test: *p* < 0.01), with the highest mortality observed in the low eGFRdiff group (Fig. 3). The number of deaths in the low, reference, and high groups were 174, 196, and 16, respectively. Similarly, cardiovascular mortality differed significantly among the eGFRdiff groups (log-rank test, *p* < 0.01), with a greater number of cardiovascular deaths in the low eGFRdiff group. The numbers of cardiovascular deaths were 53, 62, and 5 in the low, reference, and high groups, respectively (Fig. 4).

### Cox proportional hazards analysis

Cox proportional hazards models were used to examine the association between eGFRdiff and mortality. Multivariable models were adjusted for established confounders including age, sex, BMI, eGFRcr, alcohol consumption, smoking status, hypertension, diabetes, dyslipidemia, and albuminuria. Compared with the reference group, the low eGFRdiff group was independently associated with an increased risk of all-cause mortality (HR: 1.41, 95% CI: 1.10–1.80; *p* < 0.01) (Table 2). In contrast, the high eGFRdiff group was significantly associated with a decreased risk of all-cause mortality (HR: 0.56, 95% CI: 0.33–0.97; *p* = 0.04). Regarding cardiovascular mortality, no statistically significant associations were observed in either the low eGFRdiff group (HR: 1.39, 95% CI: 0.89–2.16; *p* = 0.15) or the high eGFRdiff group (HR: 0.64, 95% CI: 0.25–1.63; *p* = 0.35) (Table 3).

**Table 2:** Univariable and multivariable Cox proportional hazards analyses of all-cause mortality including eGFRdiff (eGFRcys − eGFRcr)

	Univariable analyses			Multivariable analyses		
	HR	95% CI	*P*-value	HR	95% CI	*P*-value
Age (per year increase)	1.12	1.11–1.13	< 0.01	1.10	1.08–1.12	< 0.01
Male sex	2.45	2.13–2.82	< 0.01	2.31	1.73–3.09	< 0.01
*BMI*						
< 18.5 vs 18.5–25.0	1.52	1.16–1.99	< 0.01	1.27	0.79–2.04	0.33
≥ 25.0 vs 18.5–25.0	1.03	0.89–1.20	0.66	0.90	0.71–1.14	0.39
eGFRcr (per mL/min/1.73 m^2^ increase)	0.98	0.98–0.98	< 0.01	0.99	0.98–1.00	0.01
Alcohol consumption	1.17	1.02–1.33	0.03	0.94	0.72–1.22	0.63
Smoking	1.61	1.40–1.84	< 0.01	1.32	1.02–1.70	0.03
Hypertension	1.46	1.28–1.67	< 0.01	0.98	0.79–1.21	0.85
Diabetes	2.23	1.77–2.82	< 0.01	1.76	1.23–2.53	< 0.01
Dyslipidemia	0.79	0.69–0.90	< 0.01	0.88	0.71–1.09	0.24
Albuminuria	1.98	1.69–2.31	< 0.01	1.31	1.00–1.73	0.05
*eGFRdiff (eGFRcys − eGFRcr)*						
Low (< − 10) vs (− 10 to 10)	1.50	1.22–1.84	< 0.01	1.41	1.10–1.80	< 0.01
High (≥ 10) vs (− 10 to 10)	0.29	0.17–0.48	< 0.01	0.56	0.33–0.97	0.04

*HR* Hazard ratio, *CI* Confidence interval, *BMI* Body mass index, eGFR: estimated glomerular filtration rate

**Table 3:** Univariable and multivariable Cox proportional hazards analyses of cardiovascular mortality including eGFRdiff (eGFRcys − eGFRcr)

	Univariable analyses			Multivariable analyses		
	HR	95% CI	*P*-value	HR	95% CI	*P*-value
Age (per year increase)	1.15	1.13–1.16	< 0.01	1.10	1.07–1.14	< 0.01
Male sex	2.22	1.74–2.84	< 0.01	1.88	1.10–3.21	0.02
*BMI*						
< 18.5 vs 18.5–25.0	2.00	1.28–3.12	< 0.01	1.46	0.63–3.42	0.38
≥ 25.0 vs 18.5–25.0	1.24	0.96–1.61	0.10	1.16	0.77–1.75	0.47
eGFRcr (per mL/min/1.73 m^2^ increase)	0.97	0.96–0.98	< 0.01	0.99	0.97–1.00	0.047
Alcohol consumption	1.15	0.91–1.46	0.25	0.87	0.54–1.42	0.58
Smoking	1.66	1.30–2.11	< 0.01	1.79	1.12–2.87	0.01
Hypertension	1.65	1.30–2.09	< 0.01	1.00	0.68–1.47	0.99
Diabetes	3.29	2.29–4.73	< 0.01	2.93	1.71–5.01	< 0.01
Dyslipidemia	0.68	0.54–0.87	< 0.01	0.63	0.42–0.94	0.03
Albuminuria	2.64	2.03–3.44	< 0.01	1.93	1.23–3.01	< 0.01
*eGFRdiff (eGFRcys − eGFRcr)*						
Low (< − 10) vs (− 10 to 10)	1.45	1.01–2.10	0.045	1.39	0.89–2.16	0.15
High (≥ 10) vs (− 10 to 10)	0.28	0.11–0.70	< 0.01	0.64	0.25–1.63	0.35

*HR* Hazard ratio, *CI* Confidence interval, *BMI* Body mass index, *eGFR* Estimated glomerular filtration rate

### Restricted cubic spline analysis

**Fig. 5:**
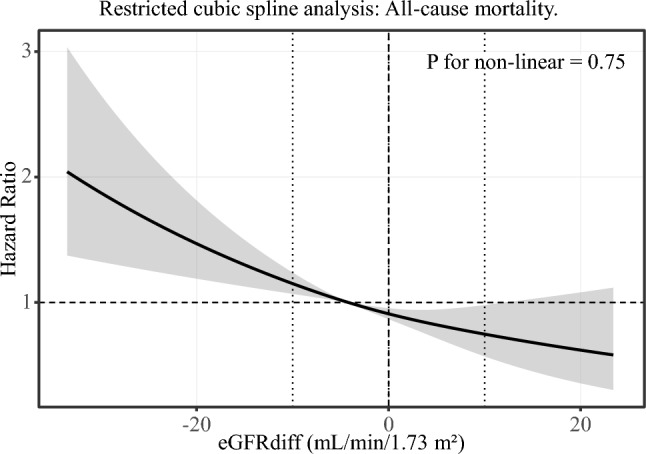
Restricted cubic spline analysis: All-cause mortality. Restricted cubic spline analysis of the association between eGFRdiff and all-cause mortality. The solid line represents the adjusted hazard ratio (HR), and the shaded area indicates the 95% confidence interval (CI). The reference value was set at eGFRdiff = 0 mL/min/1.73 m^2^. Models were adjusted for age, sex, BMI, eGFRcr, alcohol consumption, smoking status, hypertension, diabetes, dyslipidemia, and albuminuria. A monotonic increase in mortality risk was observed with decreasing eGFRdiff values. There was no statistically significant evidence of non-linearity (*p* for non-linearity = 0.75)

**Fig. 6:**
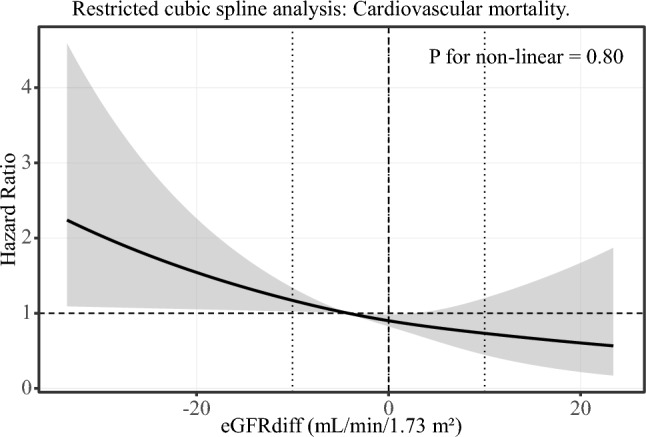
Restricted cubic spline analysis: Cardiovascular mortality. Restricted cubic spline analysis of the association between eGFRdiff and cardiovascular mortality. The solid line represents the adjusted hazard ratio (HR), and the shaded area indicates the 95% confidence interval (CI). The reference value was set at eGFRdiff = 0 mL/min/1.73 m^2^. Models were adjusted for age, sex, BMI, eGFRcr, alcohol consumption, smoking status, hypertension, diabetes, dyslipidemia, and albuminuria. Although a similar directional trend was observed, there was no statistically significant evidence of non-linearity (*p* for non-linearity = 0.80)

To further evaluate the shape of the association between eGFRdiff and mortality risk, we modeled eGFRdiff as a continuous variable using restricted cubic spline analysis within multivariable Cox proportional hazards models. The spline analysis demonstrated a monotonic increase in all-cause mortality risk with decreasing eGFRdiff values, particularly below approximately − 10 mL/min/1.73 m^2^, whereas higher eGFRdiff values were associated with lower mortality risk (Fig. 5). There was no statistically significant evidence of non-linearity in the association between eGFRdiff and all-cause mortality (*p* for non-linearity = 0.75). Similar trends were observed for cardiovascular mortality (*p* for non-linearity = 0.80, Fig. 6). The non-linearity remained non-significant even when the number of knots increased to four or five (data not shown).

### Sensitivity analysis using quartiles of eGFRdiff

To validate the robustness of our findings, we conducted a sensitivity analysis using quartiles of eGFRdiff. Participants were classified into three groups: Q1 (< − 13.79 mL/min/1.73 m^2^), Q2–Q3 (− 13.79 to 4.55 mL/min/1.73 m^2^; reference group), and Q4 (≥ 4.55 mL/min/1.73 m^2^). Compared with the reference group (Q2–Q3), Q1 was significantly associated with an increased risk of all-cause mortality (HR: 1.40, 95% CI: 1.07–1.83; *p* = 0.01). However, Q4 was not significantly associated with all-cause mortality (HR: 0.76, 95% CI: 0.55–1.04; *p* = 0.09) (Supplemental Table 1). No significant associations were observed between eGFRdiff quartiles and cardiovascular mortality. The HRs for Q1 and Q4 were 1.21 (95% CI: 0.74–1.98; *p* = 0.44) and 0.56 (95% CI: 0.30–1.04; *p* = 0.07), respectively (Supplemental Table 2).

### Adjusted survival curves

In standardized population-averaged survival curves using the same covariates as the multivariate Cox proportional hazards models, the low eGFRdiff group showed a lower adjusted survival probabilities throughout the entire period. Conversely, the high eGFRdiff group tended to have a higher adjusted survival rate. The reference eGFRdiff group was positioned in between these two groups (Supplemental figure; Likelihood ratio test: *p* < 0.01 for all-cause mortality, *p* = 0.18 for cardiovascular mortality).

### Model discrimination

We assessed whether the inclusion of eGFRdiff in the baseline risk model (which included age, sex, BMI category, eGFRcr, alcohol consumption, smoking, hypertension, diabetes, dyslipidemia, and albuminuria) improved model performance. The inclusion of eGFRdiff significantly improved the discrimination of the baseline risk model for both all-cause and cardiovascular mortality, as evaluated by IDI. The IDI values were 0.011 (95% CI: 0.004–0.017; *p* < 0.01) for all-cause mortality and 0.008 (95% CI: 0.001–0.02; *p* = 0.02) for cardiovascular mortality (Table 4).

**Table 4:** Improvement in discrimination by addition of eGFRdiff (eGFRcys − eGFRcr) to baseline model based on IDI

	All-cause mortality			Cardiovascular mortality		
	Index	95% CI	*P*-value	Index	95% CI	P-value
Baseline model + eGFRdiff	0.011	0.004–0.017	< 0.01	0.008	0.001–0.015	0.02

*IDI* Integrated discrimination improvement, CI Confidence interval, *eGFR* Estimated glomerular filtration rate

Baseline model incorporates age, male sex, BMI (< 18.5, 18.5–25.0, ≥ 25.0), eGFRcr, Alcohol consumption, smoking, hypertension, diabetes, dyslipidemia, and albuminuria

For clarity and consistency, IDI and confidence intervals were rounded to three decimal places

## Discussion

In this long-term prospective cohort study of a general Japanese population, we examined eGFRdiff and found that it was significantly associated with all-cause mortality. Specifically, individuals with lower eGFRdiff values exhibited a significantly higher risk of all-cause mortality, whereas those with higher eGFRdiff values demonstrated a lower risk, independent of other established risk factors. This association remained robust after adjusting for potential confounders. Furthermore, the inclusion of eGFRdiff in the conventional risk prediction model significantly improved its predictive performance for mortality.

These results are consistent with previous studies in Western populations, which reported that eGFRcys values lower than eGFRcr are associated with an increased risk of ESRD and mortality [16, 17]. In Japan, prior studies have reported that eGFRcys is a stronger predictor of mortality than eGFRcr [12], and that the discrepancy between the two is associated with frailty and reduced skeletal muscle mass [13, 18]. Furthermore, eGFRcys, but not eGFRcr, was associated with muscle weakness in community-dwelling Japanese older adults [19]. However, no previous study has evaluated the prognostic value of eGFRdiff in a large-scale, long-term cohort of the general Japanese population. To the best of our knowledge, this is the first such study.

Generally, cases where eGFRcr exceeds eGFRcys are thought to be caused by the overestimation of eGFRcr due to sarcopenia [5]. However, in this study cohort, baseline BMI and serum albumin levels, which serve as surrogate markers for nutritional status and muscle mass—were comparable across all eGFRdiff groups. These findings suggest that mechanisms unrelated to muscle mass may also be involved.

Notably, individuals with low eGFRdiff exhibited a higher prevalence of hypertension and elevated inflammatory markers, indicating a greater burden of vascular dysfunction and chronic inflammation. Consistent with this interpretation, prior studies have shown that the association between low eGFRcys relative to eGFRcr and adverse outcomes persists even after adjustment for baseline frailty [11]. Moreover, cystatin C is known to be influenced by non-GFR determinants such as systemic inflammation, glucocorticoid exposure, and thyroid dysfunction [20]. Collectively, these observations suggest that eGFRdiff may reflect an integrated measure of subclinical inflammation and vascular injury rather than muscle mass alone. Importantly, more than 30% of participants exhibited a low eGFRdiff, indicating that this discordance is a common phenomenon in the general population.

Strengths of our study include its large sample size, extended follow-up period, and population-based design, which enhances its external validity. Additionally, serum creatinine and cystatin C levels were measured using standardized methods, and rigorous multivariable adjustments were applied. However, this study has several limitations. First, data on frailty status, thyroid function, and systemic glucocorticoid use were not available and could not be evaluated in this study. Second, information on the prevalence of cancer at baseline was not collected, and therefore its potential influence on serum creatinine levels, eGFR estimation, and mortality risk could not be assessed. Third, eGFR was evaluated only at baseline, and changes over time were not considered. Finally, this study was conducted in a single region of Japan, and exclusion of participants without cystatin C measurements may have introduced selection bias.

In conclusion, our study demonstrated that the discrepancy between eGFRcys and eGFRcr is independently associated with all-cause mortality in the general Japanese population. Incorporating both creatinine and cystatin C measurements to calculate eGFRdiff may enable more accurate risk stratification and help identify individuals who could benefit from closer clinical follow-up and targeted interventions.

## Supplementary Information

Below is the link to the electronic supplementary material.

Supplementary file1 (DOCX 41 kb)

Standardized population-averaged survival curves for (A) all-cause mortality and (B) cardiovascular mortality according to eGFRdiff categories (< − 10, − 10 to 10 [reference], and ≥ 10 mL/min/1.73 m^2^). Survival probabilities were estimated from multivariable Cox proportional hazards models using the same covariates as in the primary analyses (age, sex, BMI, eGFRcr, alcohol consumption, smoking status, hypertension, diabetes, dyslipidemia, and albuminuria). Overall differences among the three groups were statistically significant for all-cause mortality (Likelihood ratio test, *p* < 0.01) but not for cardiovascular mortality (*p* = 0.18). Absolute differences in adjusted survival probabilities were modest throughout follow-up. Supplementary file2 (PPTX 73 kb)
