# Supplementary material for: Discrepancy between cystatin C-based and creatinine-based eGFR predicts all-cause mortality in a community-based population: the Takahata study
Source: Clin Exp Nephrol. 2026 Mar 27;30(6):883–91. doi: 10.1007/s10157-026-02853-6 (PMC13242412; doi:10.1007/s10157-026-02853-6)
Supplement: Supplementary file 1 — Supplementary file1 (DOCX 41 kb) [file 10157_2026_2853_MOESM1_ESM.docx]

Supplemental Table 1. Univariable and multivariable Cox proportional hazards analyses of all-cause mortality based on the quartiles of eGFRdiff (eGFRcys − eGFRcr).

|  | Univariable analyses | | | Multivariable analyses | | |
| --- | --- | --- | --- | --- | --- | --- |
|  | HR | 95% CI | P-value | HR | 95% CI | P-value |
| Age (per year increase) | 1.12 | 1.11–1.13 | < 0.01 | 1.10 | 1.08–1.12 | < 0.01 |
| Male sex | 2.45 | 2.13–2.82 | < 0.01 | 2.32 | 1.74–3.10 | < 0.01 |
| BMI |  |  |  |  |  |  |
| < 18.5 vs 18.5–25.0 | 1.52 | 1.16–1.99 | < 0.01 | 1.27 | 0.79–2.04 | 0.32 |
| ≥ 25.0 vs 18.5–25.0 | 1.03 | 0.89–1.20 | 0.66 | 0.90 | 0.71–1.14 | 0.39 |
| eGFRcr (per mL/min/1.73 m^2^ increase) | 0.98 | 0.98–0.98 | < 0.01 | 0.99 | 0.98–1.00 | 0.01 |
| Alcohol consumption | 1.17 | 1.02–1.33 | 0.03 | 0.93 | 0.72–1.22 | 0.61 |
| Smoking | 1.61 | 1.40–1.84 | < 0.01 | 1.34 | 1.04–1.73 | 0.02 |
| Hypertension | 1.46 | 1.28–1.67 | < 0.01 | 1.01 | 0.81–1.25 | 0.95 |
| Diabetes | 2.23 | 1.77–2.82 | < 0.01 | 1.82 | 1.27–2.61 | < 0.01 |
| Dyslipidemia | 0.79 | 0.69–0.90 | < 0.01 | 0.88 | 0.70–1.09 | 0.23 |
| Albuminuria | 1.98 | 1.69–2.31 | < 0.01 | 1.32 | 1.00–1.74 | 0.048 |
| eGFRdiff (eGFRcys − eGFRcr) |  |  |  |  |  |  |
| Q1 (< −13.79) vs Q2−Q3 (−13.79 to 4.55) | 1.31 | 1.05–1.64 | 0.02­­ | 1.40 | 1.07–1.83 | 0.01 |
| Q4 (≥ 4.55) vs Q2−Q3 (−13.79 to 4.55) | 0.46 | 0.34–0.62 | < 0.01­­ | 0.76 | 0.55–1.04 | 0.09 |
| HR: hazard ratio, CI: confidence interval, BMI: body mass index, eGFR: estimated glomerular filtration rate, | | | | | | |

Supplemental Table 2. Univariable and multivariable Cox proportional hazards analyses of cardiovascular mortality based on the quartiles of eGFRdiff (eGFRcys − eGFRcr).

|  | Univariable analyses | | | Multivariable analyses | | | |
| --- | --- | --- | --- | --- | --- | --- | --- |
|  | HR | 95% CI | P-value | | HR | 95% CI | P-value |
| Age (per year increase) | 1.15 | 1.13–1.16 | < 0.01 | | 1.10 | 1.07–1.14 | < 0.01 |
| Male sex | 2.22 | 1.74–2.84 | < 0.01 | | 1.94 | 1.14–3.32 | 0.01 |
| BMI |  |  |  | |  |  |  |
| < 18.5 vs 18.5–25.0 | 2.00 | 1.28–3.12 | < 0.01 | | 1.49 | 0.64–3.48 | 0.36 |
| ≥ 25.0 vs 18.5–25.0 | 1.24 | 0.96–1.61 | 0.10 | | 1.15 | 0.76–1.74 | 0.50 |
| eGFRcr (per mL/min/1.73 m^2^ increase) | 0.97 | 0.96–0.98 | < 0.01 | | 0.99 | 0.97–1.00 | 0.03 |
| Alcohol consumption | 1.15 | 0.91–1.46 | 0.25 | | 0.88 | 0.54–1.43 | 0.60 |
| Smoking | 1.66 | 1.30–2.11 | < 0.01 | | 1.82 | 1.14–2.91 | 0.01 |
| Hypertension | 1.65 | 1.30–2.09 | < 0.01 | | 1.04 | 0.70–1.52 | 0.86 |
| Diabetes | 3.29 | 2.29–4.73 | < 0.01 | | 2.96 | 1.73–5.06 | < 0.01 |
| Dyslipidemia | 0.68 | 0.54–0.87 | < 0.01 | | 0.63 | 0.43–0.95 | 0.03 |
| Albuminuria | 2.64 | 2.03–3.44 | < 0.01 | | 1.95 | 1.24–3.04 | < 0.01 |
| eGFRdiff (eGFRcys − eGFRcr) |  |  |  | |  |  |  |
| Q1 (< −13.79) vs Q2−Q3 (−13.79 to 4.55) | 1.03 | 0.68–1.54 | 0.89­­ | | 1.21 | 0.74–1.98 | 0.44 |
| Q4 (≥ 4.55) vs Q2−Q3 (−13.79 to 4.55) | 0.33 | 0.18–0.59 | <0.01­­ | | 0.56 | 0.30–1.04 | 0.07 |
| HR: hazard ratio, CI: confidence interval, BMI: body mass index, eGFR: estimated glomerular filtration rate, | | | | | | | |
