# Supplementary material for: Discrepancy between cystatin C-based and creatinine-based eGFR predicts all-cause mortality in a community-based population: the Takahata study
Source: Clin Exp Nephrol. 2026 Mar 27;30(6):883–91. doi: 10.1007/s10157-026-02853-6 (PMC13242412; doi:10.1007/s10157-026-02853-6)
Supplement: Supplementary file 2 — Standardized population-averaged survival curves for (A) all-cause mortality and (B) cardiovascular mortality according to eGFRdiff categories (< − 10, − 10 to 10 [reference], and ≥ 10 mL/min/1.73 m2). Survival probabilities were estimated from multivariable Cox proportional hazards models using the same covariates as in the primary analyses (age, sex, BMI, eGFRcr, alcohol consumption, smoking status, hypertension, diabetes, dyslipidemia, and albuminuria). Overall differences among the three groups were statistically significant for all-cause mortality (Likelihood ratio test, p < 0.01) but not for cardiovascular mortality (p = 0.18). Absolute differences in adjusted survival probabilities were modest throughout follow-up. Supplementary file2 (PPTX 73 kb) [file 10157_2026_2853_MOESM2_ESM.pptx]

## Slide 1
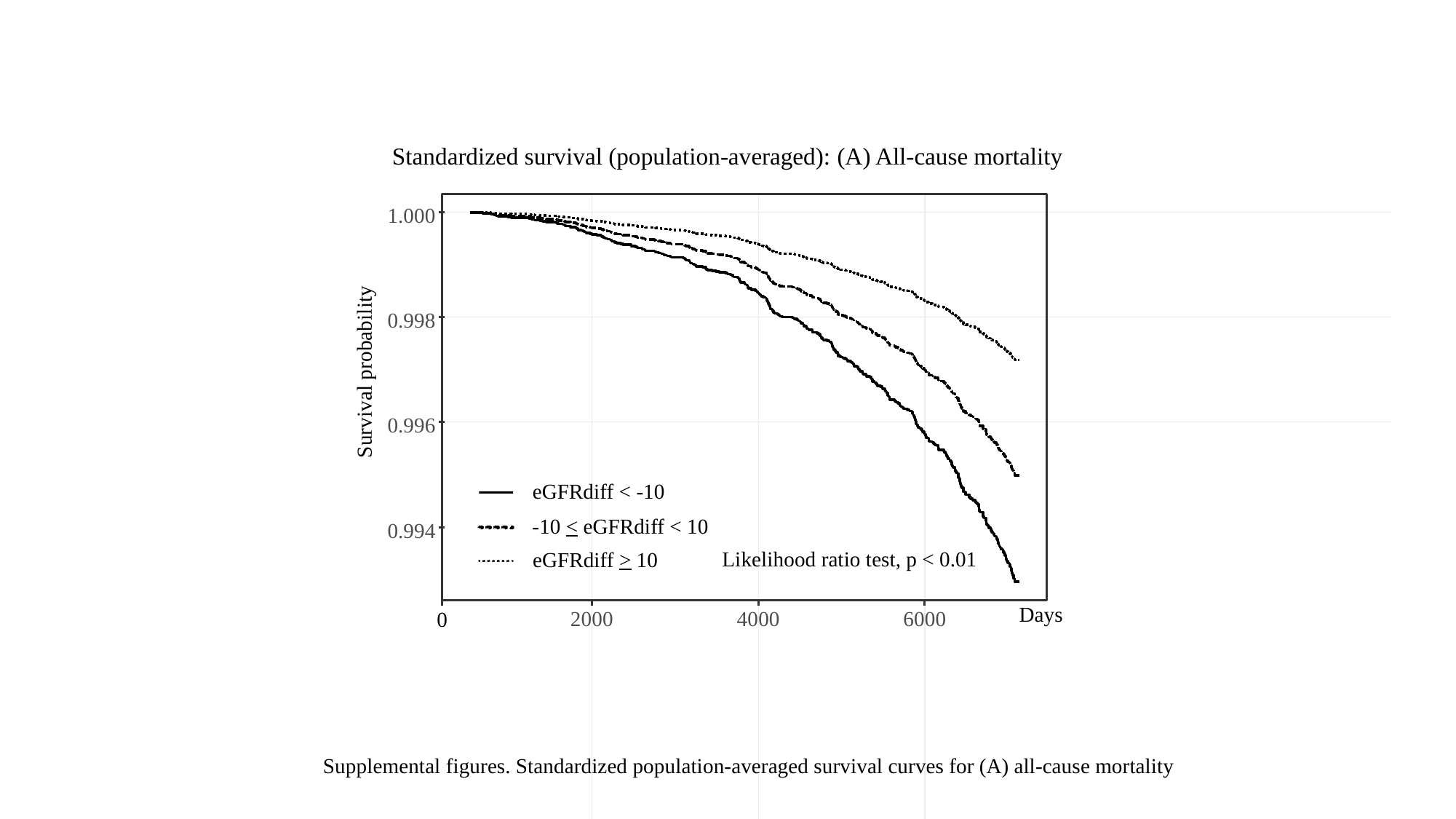

Standardized survival (population-averaged): (A) All-cause mortality
1.000
0.998
Survival probability
0.996
eGFRdiff < -10
-10 < eGFRdiff < 10
eGFRdiff > 10
0.994
Likelihood ratio test, p < 0.01
Days
0
2000
4000
6000
Supplemental figures. Standardized population-averaged survival curves for (A) all-cause mortality

## Slide 2
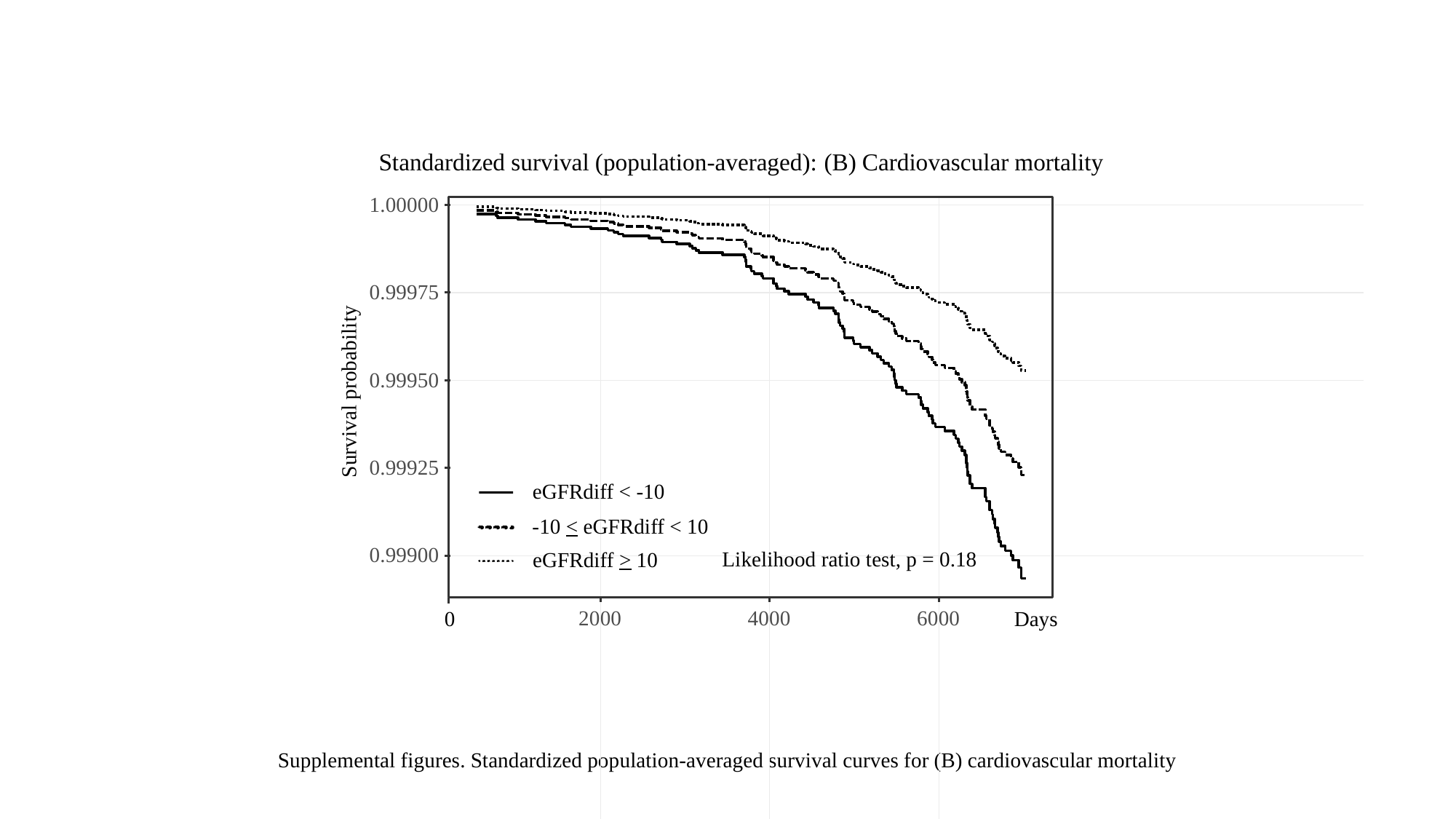

Standardized survival (population-averaged): (B) Cardiovascular mortality
1.00000
0.99975
0.99950
Survival probability
0.99925
eGFRdiff < -10
-10 < eGFRdiff < 10
eGFRdiff > 10
0.99900
Likelihood ratio test, p = 0.18
0
Days
2000
4000
6000
Supplemental figures. Standardized population-averaged survival curves for (B) cardiovascular mortality
